# Significant Clinical Associations Between Exposure Type Factors and Recurrent Wheezing and Asthma in Children

**DOI:** 10.25122/jml-2020-0143

**Published:** 2020

**Authors:** Raluca Daniela Bogdan, Lidia Rusu, Adrian Ioan Toma, Leonard Nastase

**Affiliations:** 1. Department of Pediatrics, Medicover Hospital, Bucharest, Romania; 2. Regional Center of Public Health, Iasi, Romania; 3. Department of Neonatology, Life Memorial Hospital, Bucharest, Romania; 4. Department of Medico-Surgical and Prophylactic Disciplines, Faculty of Medicine, “Titu Maiorescu” University, Bucharest, Romania; 5. Department of Neonatology, “Alessandrescu-Rusescu” National Institute of Mother and Child Health, Bucharest, Romania; 6. Department of Obstetrics-Gynecology and Neonatology, “Carol Davila” University of Medicine and Pharmacy, Bucharest, Romania

**Keywords:** Recurrent wheezing, atopy, nasopharyngeal colonisation, asthma

## Abstract

The study aimed to identify certain factors related to family history, pathological conditions, or exposure-type that are significantly correlated with recurrent wheezing and/or asthma in children. One hundred nine children with recurrent wheezing and 44 children with asthma were studied in order to identify the degree of correlation of these conditions with familial history of asthma or atopy, child’s age group, gender, premature birth, perinatal asphyxia, neonatal infection, and antibiotic treatment during the neonatal period, history of atopy and obesity and histamine intolerance, nasopharyngeal bacterial colonization, pneumonia with bronchospasm. The clinical picture of these two diseases was also compared regarding the severity of exacerbations and their response to controller therapy. The medium age of children diagnosed with recurrent wheezing was significantly lower than those diagnosed with asthma (5.64 vs. 9.01 years; p<0.001). Inside the recurrent wheezing group, age distribution differed significantly from the asthma group (p-value <0.001). Atopy was the only pathological condition significantly associated with asthma (56.0%) when compared with the recurrent wheezing group (30.2%) with a relative risk value of 1.34 (p<0.004). For patients colonized with Staphylococcus aureus, the medium number of wheezing exacerbations was significantly higher (p<0.049). Approximately 91% of patients in the recurrent wheezing group and 71% from the asthma group responded to appropriate controller treatment. Our study showed a significant association between asthma and atopy, justifying the need to monitor asthma risk in a child with wheezing and atopy. Nasal carriage of Staphylococcus aureus proved to be significantly associated with the recurrence of wheezing in children.

## Introduction

Recurrent wheezing is one of the main health problems observed during the pediatric age. According to medical data, one-third of children experience at least one wheezing episode by the age of 5. Wheezing is the main symptom in acute bronchiolitis and asthma exacerbation but is also encountered in other health conditions (so-called atypical wheezing found in tracheobronchomalacia, bronchopulmonary dysplasia, cystic fibrosis, immune deficiencies, and foreign body aspiration) [1-4].

The disease has many phenotypic variants. A large number of genetic and exposure factors have also been associated with wheezing recurrence. Tucson’s Children’s Respiratory Study enrolled 1246 subjects from 1980 till 2003, studying relevant associations between exposures and acute and chronic conditions affecting the respiratory system. The study describes several phenotypic patterns based on the empirical observation that only a minority of children presenting with recurrent wheezing continue to wheeze after 5 years and are ultimately diagnosed with asthma. The study described three major types of wheezing [5]:

1. Transient infant wheezing: it characterizes 80% of wheezing children during their first year of life, 60% of children wheezing during their second year of life, and 30% of children wheezing during their third year of life. This type is not particularly associated with atopy, family history of atopy, elevated IgE levels, eosinophilia, or atopic dermatitis. Risk factors, according to the above-mentioned study, are reduced lung function, maternal smoking during pregnancy, and the young age of the mother. In this group of children, functional residual capacity increases during the first years of life without reaching the values found in non-wheezing children. Later during adult life, these children might be prone to developing chronic obstructive pulmonary disease, especially if they become smokers, thus further narrowing their airways.
2. Non-atopic wheezing: this group comprises children continuing to wheeze after the age of 3, for which no atopy can be documented. 40% of children older than 3 years with recurrent wheezing are non-atopic. The respiratory syncytial virus (RSV) infection during infancy is among the associated risk factors, although other viruses may also be involved. A hallmark of this condition is the reversibility of bronchial obstruction to inhaled albuterol.
3. Atopic wheezing: this group comprises children who started wheezing before the age of 3, whose pulmonary function is expected to be lower at 11 years of age compared to children who started wheezing after the age of 3 [5].

Studies revealed the need for a predictive score for asthma, a concept also mentioned in the Global Initiative for Asthma (GINA) guidelines as a resource for predicting asthma in children wheezing before the age of 5. Going even further, the guidelines draw attention to the fact that these temporal and symptomatic classifications are limited by the fact that phenotypes change over time. In consequence, the need for a predictive score for asthma becomes clearer. From this aspect, wheezing for more than ten days, having more than three exacerbations per year, and associated atopy seem to be the most important predicting factors for asthma development later in life.

The guideline recommends a therapeutic trial depending on the severity and duration of symptoms using either a short-acting beta-agonist (SABA), an inhaled corticosteroid, a leukotriene inhibitor, or an association between these agents. The response to treatment enables us to anticipate asthma after performing a differential diagnosis [5, 6].

There seems to be a cluster of genetic and exposure type factors that act either directly through narrowing airways or indirectly through promoting switching from TH1 mediated inflammation to TH2 mediated response leading to atopy [7].

Starting from the Venn diagram representation of asthma at the intersection of early wheezing, late wheezing and atopy, our study aimed to identify certain factors related to family history or exposure type that are significantly correlated with recurrent wheezing and/or asthma in children. Identifying such factors will help find data for risk stratification, thus justifying the efforts to closely monitor and treat this category of children [8, 9].

## Materials and Methods

### Study group

We have studied the medical records of 153 pediatric patients from January 2016 till March 2019 and received a diagnosis either of “recurrent wheezing” - ICD code R06 - defined as 3 or more episodes of wheezing or “asthma” - ICD code J45. Seventy-two patients were excluded from the study because their medical records did not have enough evidence to support the diagnoses of either ”asthma” or “recurrent wheezing”, because these diagnoses have been excluded by clinical evolution, or because biological data proved that the wheezing had not been correctly documented in the first place.

Data on environmental exposure and family history have been collected. The study was retrospective, descriptive, searching to identify significant associations between these factors, the recurrence of wheezing and/or the diagnosis of “asthma”. The research procedure was formally approved by the Ethics Committee of the Medicover Hospital, Bucharest.

### Parameters studied

We have tried to identify the degree of correlation with the recurrence of wheezing and the risk of asthma considering familial history (first-degree relatives) of asthma or atopy, child’s age group, child’s gender, personal history regarding premature birth, perinatal hypoxic-ischemic injury, neonatal infection and antibiotic treatment during the neonatal period, personal history of atopy and other certain diseases (e.g., obesity, histamine intolerance), nasopharyngeal bacterial colonization, personal history of pneumonia with bronchospasm.

The clinical pictures of these two diseases were also compared concerning the severity of exacerbations and their response to controller therapy (inhaled corticosteroids and/or leukotriene receptor antagonists).

### Statistical analysis

Age distribution was evaluated using Skewness and Kurtosis analysis. The data proved to have a homogenous distribution around the median value, thus enabling us to apply tests to determine the statistical significance.

The association between family history, environmental exposures, and wheezing recurrence for developing asthma has been measured using specific tools for evaluating the “exposure-outcome” type of reaction that is measuring the “relative risk” and “interval of confidence”. The power of this association was measured using the “p” value (<0.05 being considered statistically significant).

## Results

### Study group characteristics

We analyzed the medical records of the 153 children who received either a diagnosis of recurrent wheezing or a diagnosis of asthma during a period of approximately 3 years (from January 2016 till March 2019). We identified two groups based on the diagnosis: group 1 included 109 children diagnosed with “asthma,” and group 2 included 44 children diagnosed with “recurrent wheezing”.

**Figure 1:**
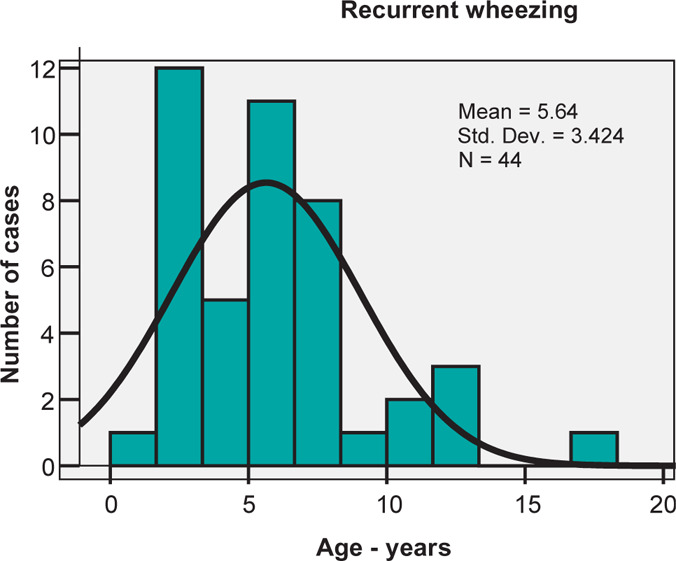
Age distribution histogram for recurrent wheezing.

Children diagnosed with recurrent wheezing had ages between 1 and 17 years with a mean age of 5.64±3.42 years and a fairly similar median value of 5 years (Figure 1).

**Figure 2:**
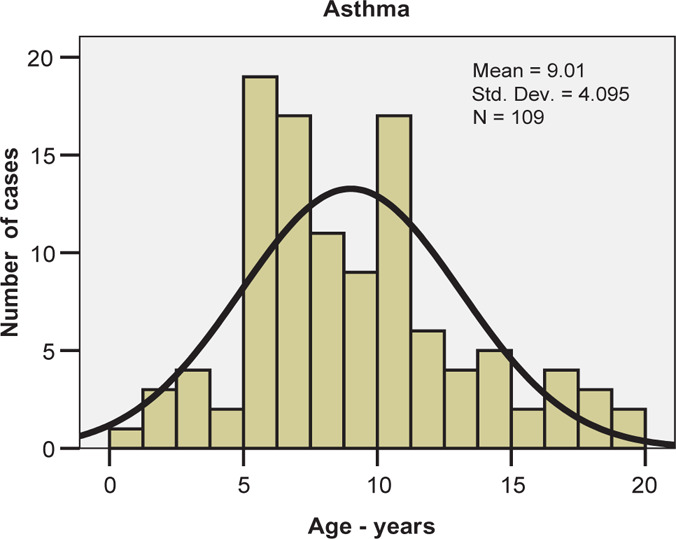
Age distribution histogram for asthma.

Patients in the asthma group had ages between 1 and 20 years with a mean value of 9.01 ± 4.10 years and a median value of 8 years – fairly similar to the medium age (Figure 2). The Skewness-Kurtosis test results indicate that age intervals are homogenously distributed among the two groups, thus allowing us to apply tests for statistical significance (Table 1).

**Table 1:** Statistical indexes of age: comparative analysis between the two study groups.

Study group	Recurrent wheezing	Asthma
**No. of recordings**	44	109
**Medium value**	5.64	9.01
**Median value**	5.00	8.00
**Standard deviation**	3.42	4.10
**Variant**	11.73	16.77
**Skewness Test**	1.236	0.618
**Skewness Test Error**	0.357	0.231
**Kurtosis Test**	1.815	0.058
**Kurtosis Test Error**	0.702	0.459
**Minimum**	1	1
**Maximum**	17	20
**Percentile**		
**25**	3	6
**50**	5	8
**75**	7	11

**Figure 3:**
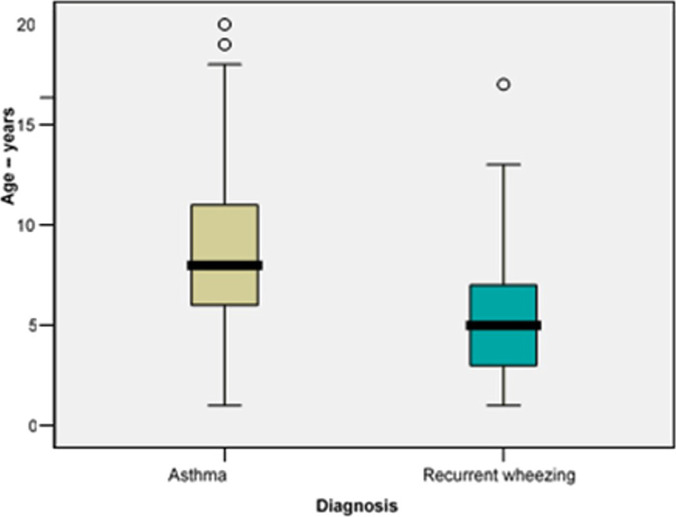
Comparison of mean age between the study groups.

The mean age of children diagnosed with recurrent wheezing proved to be significantly lower than that of children diagnosed with asthma (5.64 vs. 9.01 years; p<0.001) (Figure 3).

**Figure 4:**
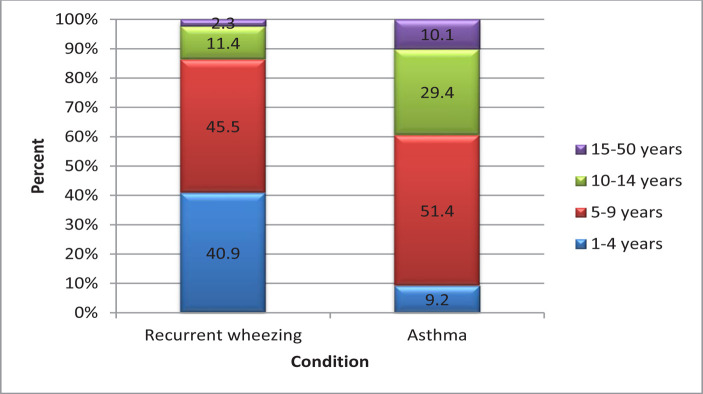
Age distribution in the studied groups.

Inside the recurrent wheezing group, age distribution differed significantly from the asthma group (p value <0.001) (Figure 4).

**Figure 5:**
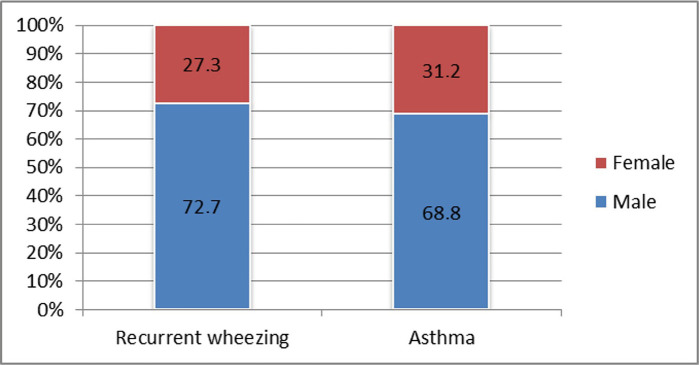
Distribution of study participants according to gender.

The male gender was predominant in both recurrent wheezing and asthma groups. Comparing the two groups, there seems to be a slight predominance of the male gender in the recurrent wheezing group. However, the difference lacks statistical significance - 72.7% vs. 68.8%; p=0.630 (Figure 5).

### Study of risk factors

**Figure 6:**
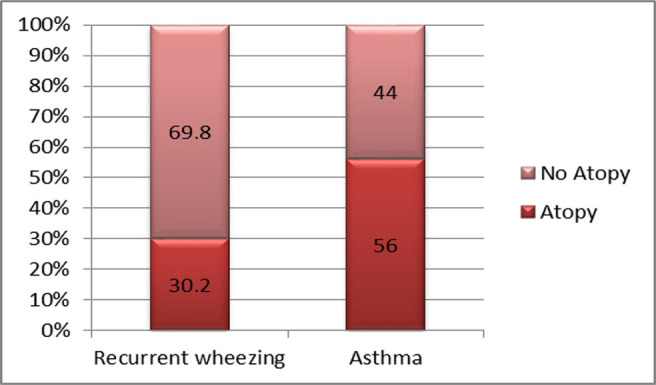
Atopy frequency among the two study groups.

**Atopy** was the only pathological condition significantly associated with asthma when compared with the recurrent wheezing group, with a relative risk (RR) value of 1.34, a confidence interval (CI) of 95%, and an incidence of 56.0% vs. 30.2%; p<0.004 (Figure 6).

**Figure 7:**
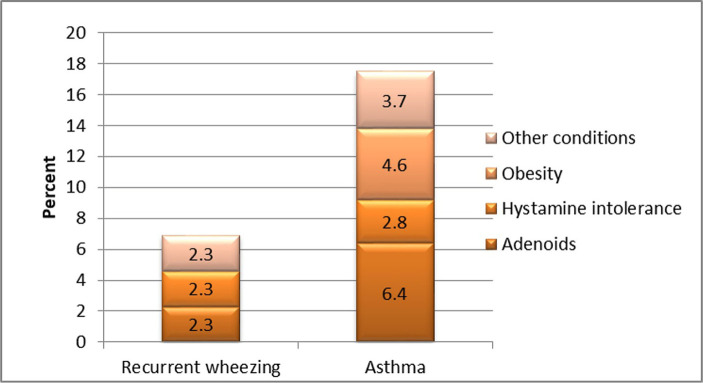
Comparative analysis of associated conditions in both study groups.

No other condition was found to be significantly associated from a statistical point of view to either recurrent wheezing or asthma when compared between study groups. Although some entities such as “adenoids”, “obesity” and “histamine intolerance” have been encountered more frequently in the asthma group; the difference lacks statistical significance - p=0.280 (Figure 7).

**Figure 8:**
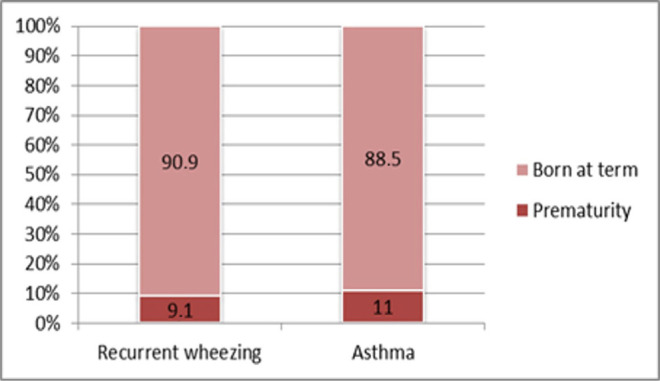
Case distribution based on the occurrence of premature birth in both study groups.

**Prematurity** did not prove to be significantly associated with recurrent wheezing or with asthma (Figure 8). Although we have found a slightly larger proportion of preemies among children diagnosed with asthma, the correlation lacked statistical significance (9.1% vs. 11%; p=0.718).

No statistically significant differences among study groups have been found regarding **perinatal hypoxic injury** (9.1% vs. 5.8%; p=0.718). However, the estimated relative risk proved to be 1.32 times higher for recurrent wheezing compared to asthma (RR=1.32; CI 95%: 0.56-3.08). The small number of cases might be responsible for failing to identify a difference that is statistically significant.

**Infection during the neonatal period** seems to be more frequently associated with asthma, with a relative risk that is 1.66 times higher when compared to the recurrent wheezing group (RR=1.66; CI95%: 1.39-1.98). However, due to the low number of cases, the association lacks statistical significance (3.8% vs. 0%; p=0.158) (Figure 10).

**Figure 9:**
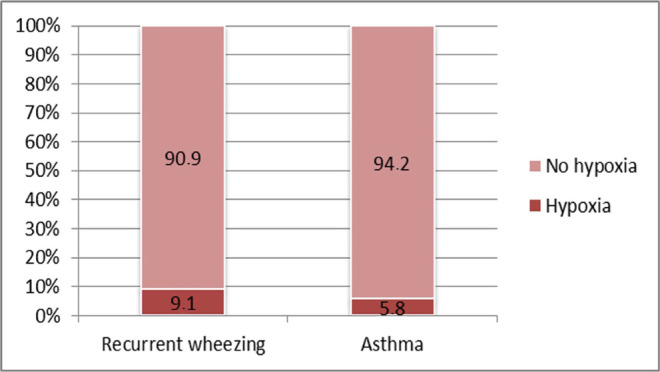
Case distribution based on the occurrence of perinatal hypoxic injury in both study groups.

**Figure 10:**
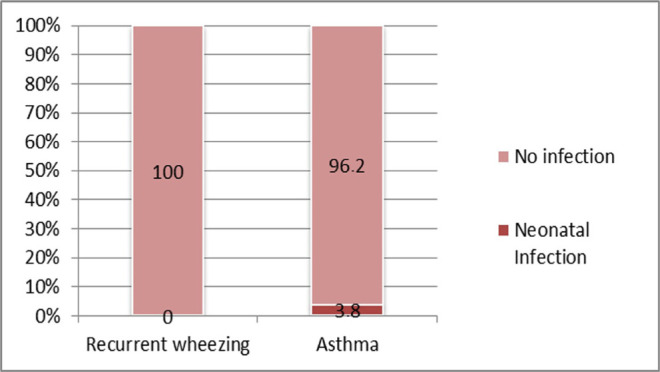
Case distribution based on the occurrence of infections in the neonatal period between the study groups.

**Figure 11:**
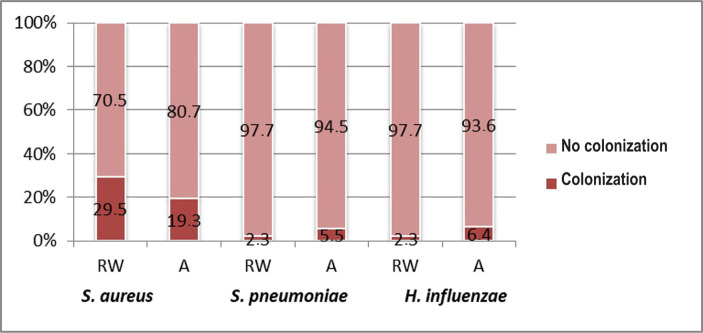
Comparative analysis of nasopharyngeal colonization in both study groups (RW – recurrent wheezing; A – asthma).

**Nasopharyngeal colonization** with *Staphylococcus aureus* seemed to be a risk factor for recurrence of wheezing (RR=1.47; IC95%: 0.87-2.48; p=0.174) while nasopharyngeal carriage of *Streptococcus pneumoniae* (RR=1.22; CI95%: 0.88-1.67; p=0.355) or *Haemophilus influenzae* (RR=1.24; CI95%: 0.94-1.65; p=0.260) seem to have been more frequently associated with asthma although the association lacks statistical significance due to the low number of cases (Figure 11).

**Figure 12:**
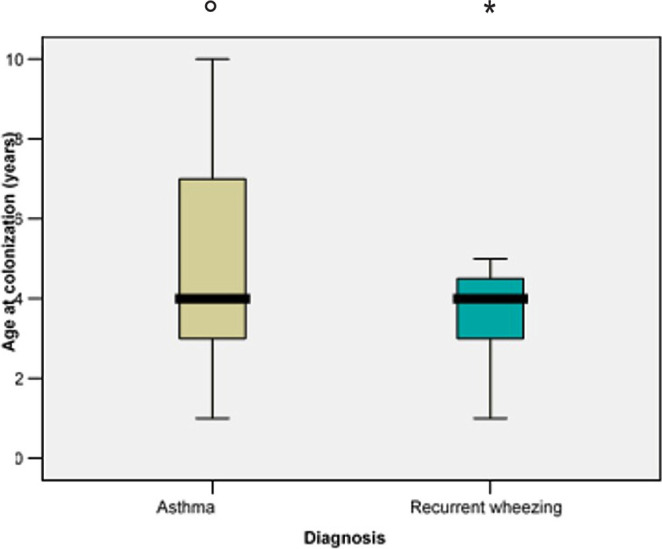
Medium age for nasopharyngeal colonization in both groups.

The medium age upon colonization did not differ significantly for patients diagnosed with recurrent wheezing when compared to asthma (4.42 vs. 5.20 years; p=0.528) (Figure 12).

**Figure 13:**
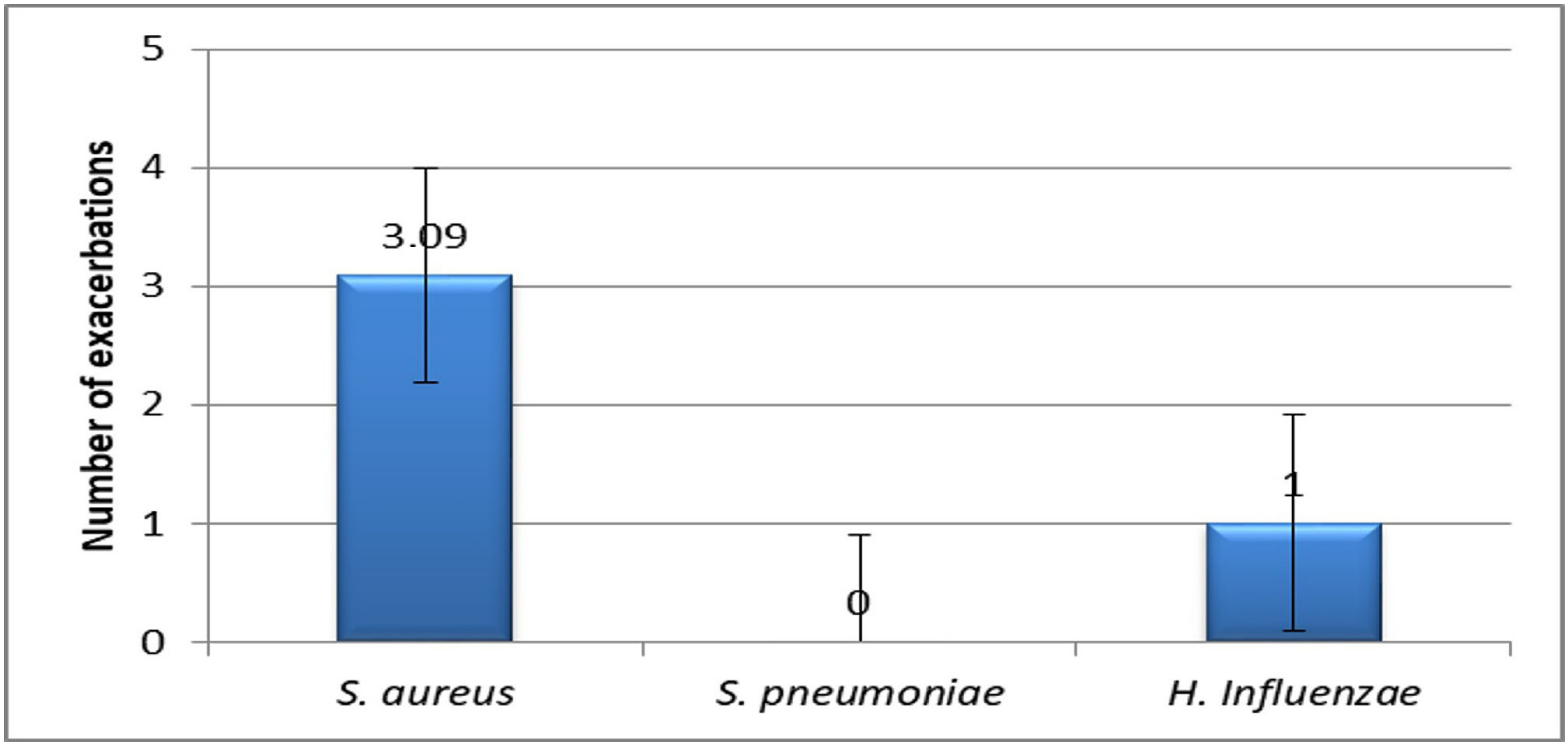
Medium number of wheezing exacerbations for different nasopharyngeal bacterial colonisations.

For patients **colonized with *Staphylococcus aureus***, the medium number of wheezing exacerbations was significantly higher (p<0.049) (Figure 13), thus demonstrating that *Staphylococcus aureus* colonization constitutes a risk factor for the recurrence of wheezing.

**Figure 14:**
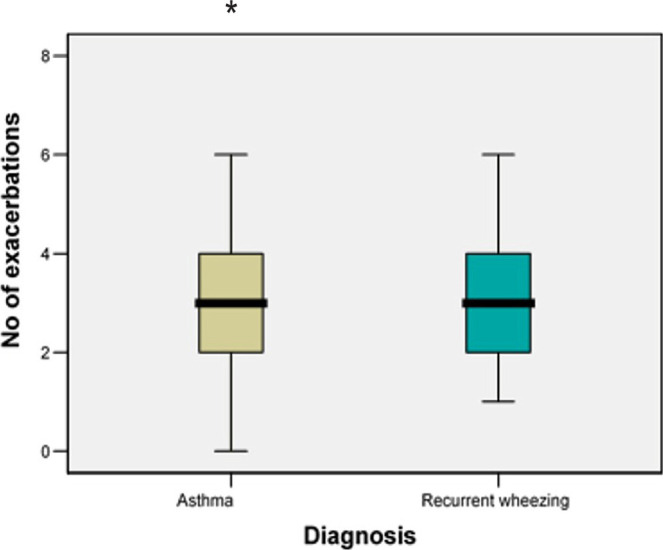
Medium number of exacerbations in both study groups.

Regarding the **medium number of exacerbations**, there was no statistically significant difference between the asthma group - 3.06±2.15 and recurrent wheezing group - 2.92±1.44 (p=0.831) (Figure 14).

**Figure 15:**
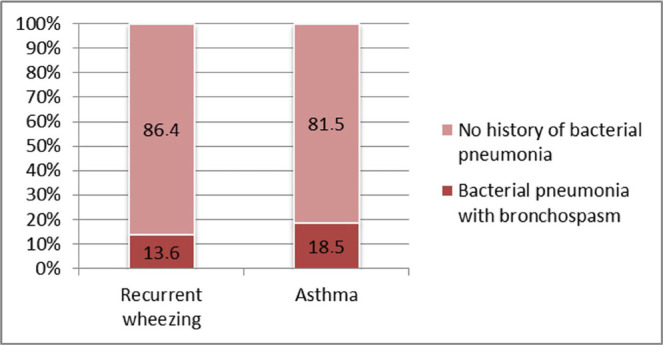
Case distribution based on bacterial pneumonia with bronchospasm history in the recurrent wheezing vs. asthma group.

**Personal history of bacterial pneumonia with bronchospasm** has been more frequently associated with asthma, but the association lacks statistical significance (18.5% vs. 13.6%; p=0.460) (Figure 15).

### Evolution and response to therapy

**Figure 16:**
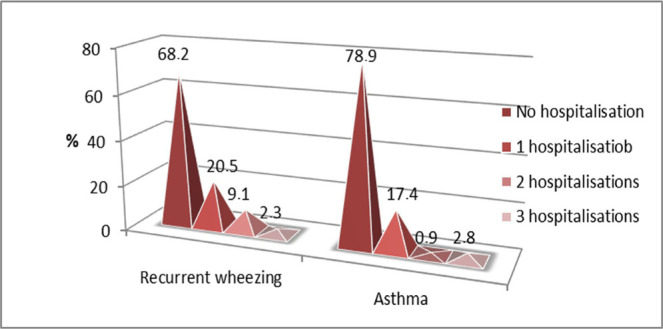
Case distribution for severe exacerbations requiring hospitalization in the two study groups.

Regarding the number of hospitalizations for severe episodes of bronchospasm, the result of the Kruskal Wallis test showed no significant differences between the two study groups - p=0.097(Figure 16).

**Figure 17:**
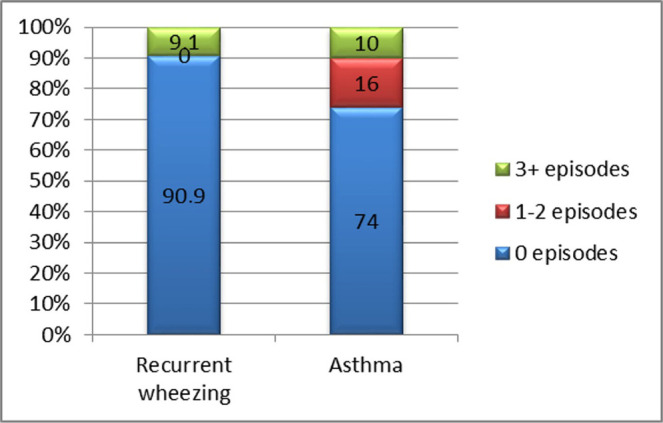
Case distribution based on the clinical response to controller treatment: comparative analysis in both study groups.

Case distribution of exacerbations under properly administered controller treatment (leukotriene inhibitors and/or inhaled corticosteroids) showed that approximately 91% of patients from the “recurrent wheezing” group and 71% from the “asthma” group responded to treatment. The difference between study groups was not statistically significant (p=0.488) (Figure 17).

## Discussion

The strong points of our study are represented by the relative homogeneity regarding the socio-economic status, place of living and medical care due to common protocols of childcare within our study groups. Both group’s homogeneity in terms of age group distribution makes them appropriate for applying statistical significance tests [12]. As weak points of the research, we could identify the following: the relatively small number of patients that was probably responsible for the lack of statistical significance for some of our data and the retrospective type of research that rendered us unable to document some exposure type factors for all our patients.

The medium age of children diagnosed with recurrent wheezing was found to be lower than that of children diagnosed with asthma, which correlates with information found in the medical literature, being explained by the well-known fact that recurrent wheezing occurs in younger children and also by the fact that “recurrent wheezing” is the diagnosis chosen by doctors for a wheezing child who is too young to be able to document well atopy, bronchial responsiveness and other factors contributing to the diagnosis of asthma [10, 11].

The male gender seemed to be more prone to recurrent wheezing and asthma diagnosis, being more than twice the number of females in both study groups. Regarding gender preponderance, no statistical differences were found among the study groups. Thus, we can conclude that this criterion cannot be used as a prognostic factor for evolution to asthma. Our results are similar to the results found in other papers. Other studies have found equal distributions among sexes, the difference resulting in clinical manifestations, and the presence of certain risk factors [13, 14]. We can speculate that study group dimensions and geographical area influenced that result.

Regarding pathologic conditions associated with the diagnosis of asthma or recurrent wheezing, the association with atopy proved to be a statistically significant risk factor for asthma. This association has been proven to be statistically significant in other medical papers. Although other diseases have been found in different proportions in both study groups (e.g., adenoids, histamine intolerance, obesity), the associations were not statistically significant, although some literature data speak of an association between obesity and the diagnosis of asthma [16].

Neither prematurity nor perinatal hypoxia, conditions that are known to impede pulmonary maturation, have been significantly associated with asthma or recurrent wheezing. Neonatal infection was not significantly associated with either one of the conditions studied. However, this condition came closest to achieving statistical significance in relation to asthma diagnosis, probably due to antibiotic treatment influencing microbiome formation, thus shifting the adaptative immune response from Th1 to Th2 type [17]. Prematurity has been proven by previous studies to be significantly associated with recurrent wheezing, but this association did not prove to work both ways according to our study results, showing that recurrent wheezing is a multifactorial condition for which prematurity plays only a minor role [18].

*Staphylococcus aureus* nasopharyngeal colonization proved, according to our study results, to predispose to the recurrent wheezing, an observation that is similar to the results of other studies [19].

Colonization of the nasopharynx by other bacteria (*Streptococcus pneumoniae, Haemophilus influenzae*) has been associated with asthma. Several data from the medical literature show an association between nasopharyngeal colonization and recurrent wheezing or asthma, although they admit that the degree of correlation and exact physiopathological mechanism of this association needs further studying [20, 21].

No significant differences were found neither in the frequency nor severity of exacerbations between recurrent wheezing and asthma groups, thus suggesting the equal importance of triggers and inflammatory response in both study groups. Also, we found no differences between study groups regarding bacterial pneumonia as a potential trigger.

Controller treatment proved to be efficacious in controlling exacerbations in both study groups with a slight prevalence in the recurrent wheezing group – the difference lacked statistical significance thought. Studies continue to debate the role of inhaled corticosteroids, especially regarding safety profile and posology [22].

## Conclusions

We found two types of associations with implications for clinical practice. Two categories of risk factors were identified: factors related to the apparition of a certain condition and factors that could aggravate a pre-existing disease. First, the risk of asthma was found to be significantly related to the presence of atopy in children diagnosed with wheezing. This finding could help in the early identification of children at risk, allowing for prevention strategies focused on close monitoring and early intervention. Second, the nasal carriage of *Staphylococcus aureus* represented a risk factor for the exacerbations of recurrent wheezing. Thus, close monitoring and early identification of this risk factor could decrease the number of exacerbations of this condition and a better evolution. We believe that the results of this study offer clues for an evidence-based approach for practical interventions to the early identification and reduction of the severity of the above-mentioned conditions.

When dealing with complex etiopathological and clinical entities, one needs to keep an open mind in diagnosing asthma when trying to prevent recurrent wheezing and searching and treating conditions that trigger wheezing. A supporting fact in this aspect is the modest sensibility of asthma predicting scores despite a good specificity. When managing a wheezing child, proper treatment and prompt recognition and treatment of clinical triggers are essential for controlling exacerbation of wheezing and good long-term management of asthma symptoms. Based on our findings, we recommend close surveillance of children with wheezing and atopy in order to identify early those cases progressing to asthma. Also, *Staphylococcus aureus* carriers should be monitored and treated in order to prevent wheezing episodes in patients at risk.

## Conflict of Interest

The authors declare that there is no conflict of interest.
